# Simulated clinical deployment of fully automatic deep learning for clinical prostate MRI assessment

**DOI:** 10.1007/s00330-020-07086-z

**Published:** 2020-08-07

**Authors:** Patrick Schelb, Xianfeng Wang, Jan Philipp Radtke, Manuel Wiesenfarth, Philipp Kickingereder, Albrecht Stenzinger, Markus Hohenfellner, Heinz-Peter Schlemmer, Klaus H. Maier-Hein, David Bonekamp

**Affiliations:** 1 Division of Radiology, German Cancer Research Center (DKFZ), Im Neuenheimer Feld 280, 69120 Heidelberg, Germany; 2 Department of Radiology, Affiliated Hospital of Guilin Medical University, Guangxi Guilin, People’s Republic of China; 3 Department of Urology, University of Heidelberg Medical Center, Heidelberg, Germany; 4 Division of Biostatistics, German Cancer Research Center (DKFZ), Heidelberg, Germany; 5 Department of Neuroradiology, University of Heidelberg Medical Center, Heidelberg, Germany; 6 Institute of Pathology, University of Heidelberg Medical Center, Heidelberg, Germany; 7 German Cancer Consortium (DKTK), Heidelberg, Germany; 8 Medical Image Computing, German Cancer Research Center (DKFZ), Heidelberg, Germany

**Keywords:** Prostate cancer, Magnetic resonance imaging, Artificial intelligence, Deep learning, Decision support systems, clinical

## Abstract

**Objectives:**

To simulate clinical deployment, evaluate performance, and establish quality assurance of a deep learning algorithm (U-Net) for detection, localization, and segmentation of clinically significant prostate cancer (sPC), ISUP grade group ≥ 2, using bi-parametric MRI.

**Methods:**

In 2017, 284 consecutive men in active surveillance, biopsy-naïve or pre-biopsied, received targeted and extended systematic MRI/transrectal US-fusion biopsy, after examination on a single MRI scanner (3 T). A prospective adjustment scheme was evaluated comparing the performance of the Prostate Imaging Reporting and Data System (PI-RADS) and U-Net using sensitivity, specificity, predictive values, and the Dice coefficient.

**Results:**

In the 259 eligible men (median 64 [IQR 61–72] years), PI-RADS had a sensitivity of 98% [106/108]/84% [91/108] with a specificity of 17% [25/151]/58% [88/151], for thresholds at ≥ 3/≥ 4 respectively. U-Net using dynamic threshold adjustment had a sensitivity of 99% [107/108]/83% [90/108] (*p* > 0.99/> 0.99) with a specificity of 24% [36/151]/55% [83/151] (*p* > 0.99/> 0.99) for probability thresholds d3 and d4 emulating PI-RADS ≥ 3 and ≥ 4 decisions respectively, not statistically different from PI-RADS. Co-occurrence of a radiological PI-RADS ≥ 4 examination and U-Net ≥ d3 assessment significantly improved the positive predictive value from 59 to 63% (*p* = 0.03), on a per-patient basis.

**Conclusions:**

U-Net has similar performance to PI-RADS in simulated continued clinical use. Regular quality assurance should be implemented to ensure desired performance.

**Key Points:**

*• U-Net maintained similar diagnostic performance compared to radiological assessment of PI-RADS ≥ 4 when applied in a simulated clinical deployment.*

*• Application of our proposed prospective dynamic calibration method successfully adjusted U-Net performance within acceptable limits of the PI-RADS reference over time, while not being limited to PI-RADS as a reference.*

*• Simultaneous detection by U-Net and radiological assessment significantly improved the positive predictive value on a per-patient and per-lesion basis, while the negative predictive value remained unchanged.*

**Electronic supplementary material:**

The online version of this article (10.1007/s00330-020-07086-z) contains supplementary material, which is available to authorized users.

## Introduction

In recent years, there is highest evidence that prostate MRI improves the detection of clinically significant prostate cancer (sPC) by identifying targets for subsequent biopsy, while reducing the number of biopsy cores required for appropriate sPC diagnosis [1–5]. Prostate MRI is becoming increasingly integrated into the diagnostic pathway [5] and increasingly standardized, most recently by the Prostate Imaging Reporting and Data System (PI-RADS) version 2.1 [6]. There is continued need to improve work efficiency and minimize inter-reader variability [7–9]. Artificial intelligence (AI) has the potential to make the radiological workflow more efficient, thereby reducing cost and by providing diagnostic support as well as a safety net, e.g., in the form of a virtual second reader. We have recently developed and validated a deep learning model based on the U-Net [10] architecture that demonstrated comparable performance to clinical radiological assessment [11]. The algorithm was trained using data from 250 men and validated on data from 62 men for use at our main institutional MRI scanner. After establishing the system, its clinical utility should be evaluated by continued clinical application in consecutive patients, to gain further insights into important aspects of AI deployment into clinical practice.

We hypothesized that the validated system should maintain its performance in the clinical environment for which it was developed. The purpose of the present study was to simulate continued clinical use and regular quality assurance cycles in the deployment of the previously developed U-Net for fully automatic assessment of prostate MRI images.

## Materials and methods

This retrospective analysis was performed in a previously unreported cohort of men undergoing MRI–transrectal US (MR/TRUS) fusion biopsy. The institutional ethics committee approved the study and waived written informed consent (S-156/2018) to allow analysis of a complete consecutive cohort. All men had clinical indication for biopsy based on prostate-specific antigen (PSA) elevation, clinical examination, or participation in our active surveillance program; were biopsied between January 2017 and December 2017; and were included if they met the following criteria: (a) imaging performed at our main institutional 3-T MRI system and (b) MRI/TRUS-fusion biopsy performed at our institution. Exclusion criteria were (a) history of treatment for prostate cancer (antihormonal therapy, radiation therapy, focal therapy, prostatectomy); (b) biopsy within 6 months prior to MRI; and (c) incomplete sequences or severe MRI artifacts. sPC was defined as International Society of Urological Pathology (ISUP) grade ≥ 2 [12]. Details on image preprocessing are given in Supplement S1.

### MRI protocol

T2-weighted, diffusion-weighted (DWI), and dynamic contrast-enhanced MRI were acquired on a single 3-T MRI system (Prisma, Siemens Healthineers) in accordance with European Society of Urogenital Radiology guidelines, by using the standard multichannel body coil and integrated spine phased-array coil. The institutional prostate MRI protocol is given in Supplementary Table 1.

### PI-RADS assessment

PI-RADS interpretation of mpMRI was performed by 8 board-certified radiologists during clinical routine (using PI-RADS version 2) [13], with 85% of the studies being interpreted by radiologists with at least 3 years of experience in prostate MRI. For quality assurance, prior to biopsy, all examinations were reviewed in an interdisciplinary conference and radiologists participated in regular retrospective review of MRI reports and biopsy results.

### MRI/TRUS-fusion biopsies

All men underwent grid-directed transperineal biopsy under general anesthesia using rigid or elastic software registration (BiopSee, MEDCOM and UroNav, Philips Invivo, respectively). First, MRI-suspicious lesions received fusion-targeted biopsy (FTB) (inter-quartile range (IQR) 3–5 cores, median 4 per lesion), followed by systematic saturation biopsy (22–27 cores, median 24 cores), as previously described [14, 15]. This combined biopsy approach of FTBs and transperineal systematic saturation biopsies (SBs) has been validated against and its concordance with radical prostatectomy (RP) specimen has been confirmed [15]. A median of 32 biopsies (IQR 28–37) were taken per patient, with the number of biopsies adjusted to prostate volume [16]. Histopathological analyses were performed under supervision of one dedicated uropathologist (A.S., 17 years of experience) according to the International Society of Urological Pathology standards.

### Lesion segmentation

Lesion segmentation was retrospectively performed based on clinical reports and their accompanying sector map diagrams by one investigator (X.W.), a board-certified radiologist with 5 years of experience in body imaging and 6 months of focused expertise in prostate MRI under supervision and in consensus with a board-certified radiologist (D.B.) with 11 years of experience in prostate MRI interpretation, using the polygon tool from open-source MITK software (www.mitk.org, version 2018.04) to draw the three-dimensional volumes of interest (VOI) separately on axial T2-weighted and apparent diffusion coefficient (ADC)/DWI images.

### Application of deep learning algorithm

The previously trained and validated two-dimensional 16-member U-Net ensemble [10] utilizes T2-weighted, *b*-value 1500 s/mm^2^ and ADC maps to classify each voxel as either tumor, normal-appearing prostate, or background. For each U-Net in the ensemble, output probabilities for the three classes sum up to one per voxel. The ensemble probability map is the mean of the ensemble member U-Net probability maps. For each examination, the ensemble was applied to each of the rigid, affine, and b-spline registration schemes and the map with the highest tumor probability used for further processing. Deep learning was implemented in PyTorch (version 1.2.0; https://pytorch.org) [17].

### Combined histopathological mapping

To utilize all available histopathological information including that of sPC outside of PI-RADS lesions, sextant-specific systematic and targeted lesion histopathology were fused into a combined histological reference (Supplementary Material S-2).

### Threshold adjustment and statistical analysis

Receiver operating characteristic (ROC) curves were calculated from U-Net probability predictions. U-Net probability thresholds yielding patient-based working points most closely matching PI-RADS ≥ 3 and ≥ 4 performance were obtained as outlined in Supplementary Material S-3. For application to the current cohort, three U-Net thresholds were determined: *fixed*, *dynamic*, and *limit*. *Fixed thresholds* represent the most straightforward application of the published U-Net to new examinations and are determined from the 300 most recent examinations of the published cohort. *Dynamic thresholds* are readjusted in regular intervals to keep U-Net and PI-RADS closely matched on the most recent examinations. These are initially set to the values of the fixed thresholds, applied to the 50 following examinations, then repeatedly readjusted using the most recent 300 examinations. Each patient is evaluated in a simulated prospective manner using only the dynamic threshold resulting from the most recent adjustment. *Limit thresholds* represent the theoretical limit of best dynamic threshold performance by producing the closest possible match between U-Net and PI-RADS performance and are determined from the current cohort. Only *fixed* and *dynamic* thresholds can be applied prospectively to new patients, while *limit* thresholds are an *a posteriori* reference to judge the success of threshold selection.

Sensitivity, specificity, and positive and negative predictive value were calculated and compared using the McNemar test [18]. We examined the effect of co-occurrent detection of sPC-positive men, biopsy sextants, and PI-RADS lesions by U-Net and radiologists on the positive (PPV) and negative predictive value (NPV) using a test based on relative predictive values implemented in the R package DTComPair [19, 20]. Statistical analyses were implemented in Python (Python Software Foundation, version 3.7.3, http://www.python.org) and R (R version 3.6.0, R Foundation for Statistical Computing) with details given in Supplementary Material S-4. A *p* value of 0.05 or less was considered statistically significant. All *p* values were adjusted for multiple comparisons using Holm’s method [21]. We used the Dice coefficient [22], a commonly used spatial overlap index, to compare manual and U-Net-derived lesion segmentations separately for DWI, T2w, and their combination. The mean Dice coefficient was calculated from all biopsy sPC–positive clinical lesions and U-Net-derived lesions (Supplementary Material S-5).

## Results

### Study sample characteristics

**Fig. 1:**
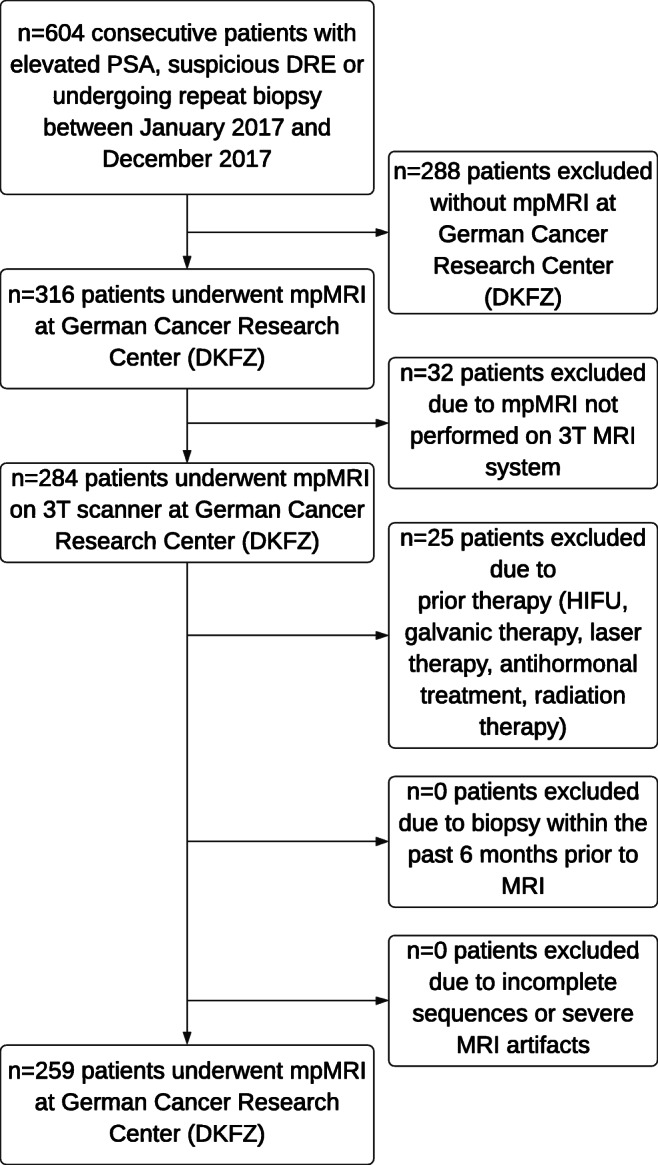
Diagram shows inclusion of patients into the study. PSA = prostate-specific antigen, mpMRI = multiparametric MRI, HIFU = high-intensity focused ultrasound

Of 604 men who presented to our institution during the inclusion period, 259 men (median age 64 [IQR61–72]) met the inclusion and exclusion criteria (Fig. 1). Demographic data and patient characteristics are shown in Table 1.

**Table 1:** Demographic and clinical characteristics of 259 included men

Cohort	*n* = 259
Age (years)	
Median (IQR)	67 (61–72)
PSA (ng/ml) median (IQR)	7.2 (5.2–10.0)
PSA density median (IQR)	0.14 (0.10–0.22)
Highest ISUP grade group (*n* (%))	
No PC	105 (40%)
I	46 (18%)
II	66 (25%)
III	18 (7%)
IV	7 (3%)
V	17 (7%)
MRI index lesion per patient (*n* (%))	
No lesion	20 (8%)
PI-RADS 2	7 (3%)
PI-RADS 3	78 (30%)
PI-RADS 4	104 (40%)
PI-RADS 5	50 (19%)
MRI assessment per lesion (*n* (%))	
Total	420 (100%)
PI-RADS 2	17 (4%)
PI-RADS 3	180 (43%)
PI-RADS 4	170 (40%)
PI-RADS 5	53 (13%)

Abbreviations: *PSA* prostate-specific antigen, *IQR* interquartile range, *ISUP* International Society of Urological Pathology, *PC* prostate cancer, *MRI* magnetic resonance imaging, *PI-RADS* Prostate Imaging Reporting and Data System

Two hundred fifty-nine men harbored 420 lesions, 299 of 420 (71%) lesions were localized in the peripheral zone and 121 of 420 (29%) lesions were localized in the transition zone. Seventeen of 420 (4%) lesions were of PI-RADS category 2, 180 of 420 (43%) of PI-RADS category 3, 170 of 420 (40%) of PI-RADS category 4, and 53 of 420 (13%) of PI-RADS category 5. In total, 112 of 420 (27%) lesions were found positive for sPC at fusion biopsy, 93 of 112 (83%) sPC-positive lesions were localized in the peripheral zone, and 19 of 112 (17%) sPC-positive lesions were localized in the transition zone. One hundred forty-five of 259 (56%) patients were biopsy-naïve, 55 of 259 (21%) patients were previously biopsied, and 59 of 259 (23%) patients participated in the active surveillance program.

#### Comparison of U-Net performance using fixed and dynamic thresholds

**Fig. 2:**
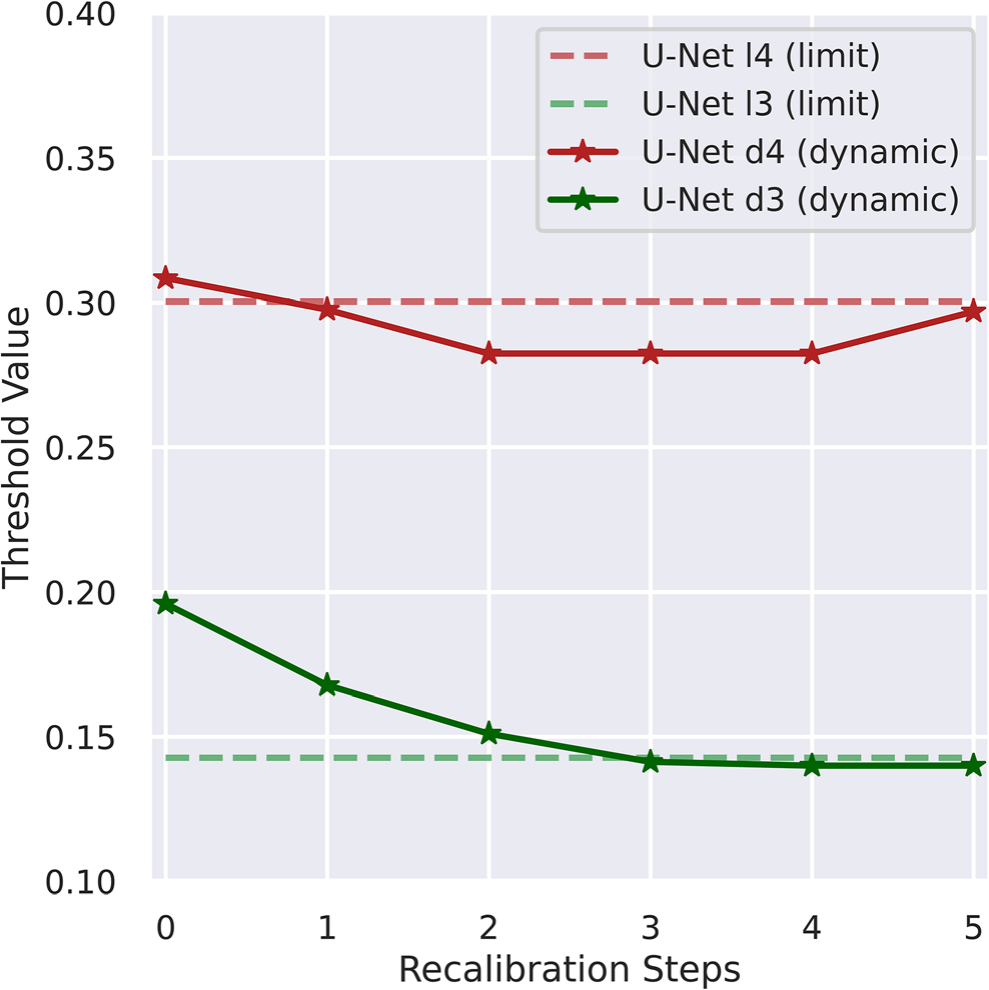
Individual dynamic thresholds over time. Initial points are equal to fixed thresholds while final points closely model limit thresholds (they do not derive from the last batch that is still being predicted with the previous thresholds). d3/l3 = dynamic/limit threshold to match clinical performance at PI-RADS greater than or equal to 3; d4/l4 = dynamic/limit threshold adjusted to match clinical performance at PI-RADS greater than or equal to 4

**Fig. 3:**
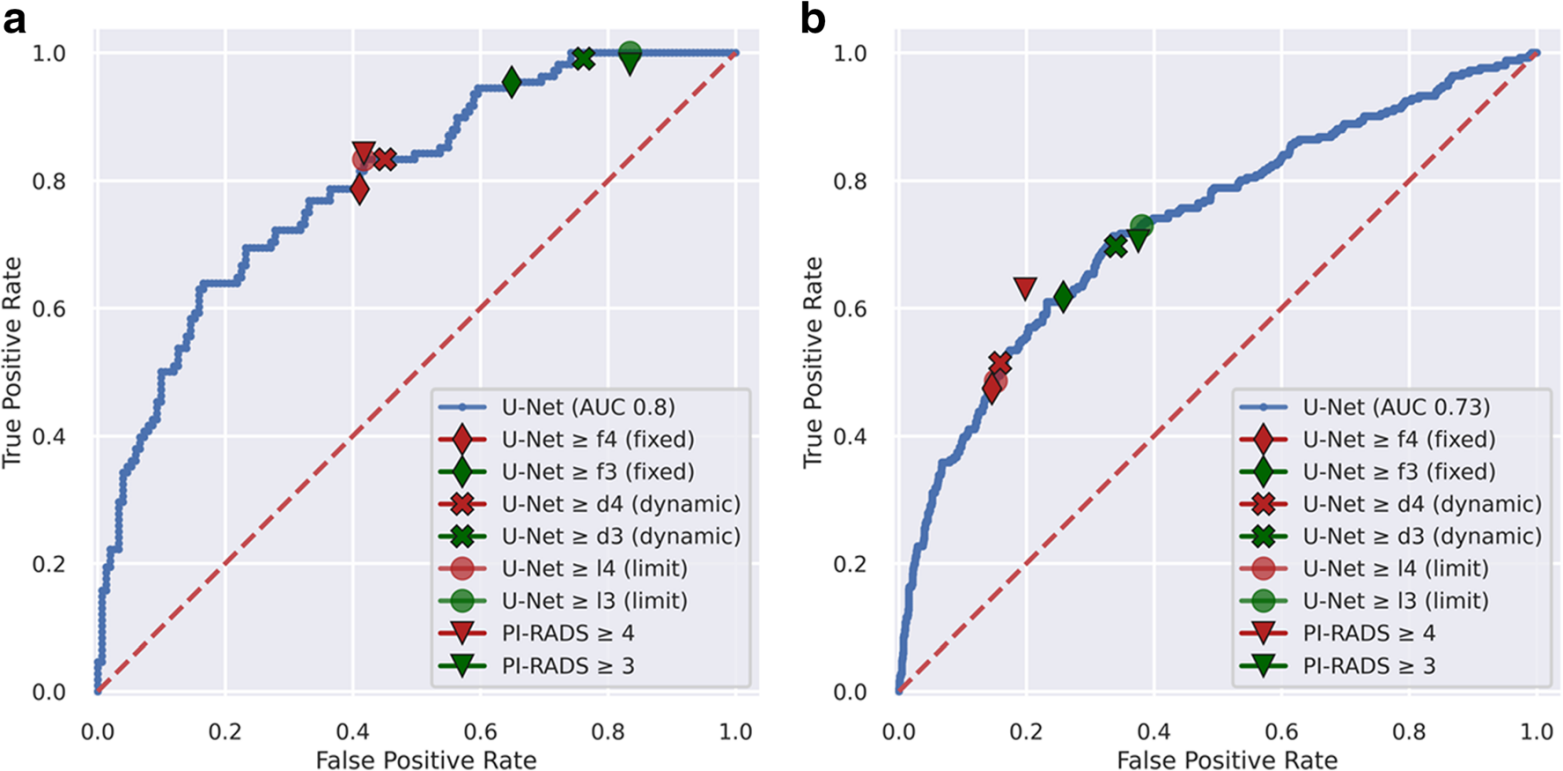
Graphs show receiver operating characteristics (ROC) curves on a per-patient (**a**) and on a per-sextant (**b**) basis for U-net performance (blue curves). Radiologist performance at Prostate Imaging Reporting and Data System (PI-RADS) cut-offs is indicated by triangles (green ≥ 3, red ≥ 4). On a patient basis, PI-RADS operating points lie very close to the blue ROC curve, indicating absence of deterioration of the U-Net model when applied to the new cohort. Three sets of U-Net probability thresholds were determined by matching PI-RADS and U-Net performance on different sets of examinations and applied to obtain working points on the ROC curves. *Fixed thresholds* determined using the 300 most recent examinations of the previously published model building cohort (diamonds); *dynamic thresholds* (crosses) are initially equal to the fixed thresholds, with each threshold derived from the previous 300 examinations and applied to the subsequent 50 examinations, until the entire current cohort is predicted; *limit thresholds* determined using all examinations of the current cohort (circles). Fixed, dynamic, and limit thresholds yield very similar working points for the PI-RADS ≥ 4 decision on the patient-based ROC curves (**a**), confirming stability of U-Net at this decision threshold. Dynamic threshold adjustment is advantageous for performance comparison at PI-RADS ≥ 3, as the resulting working point closely approximates the PI-RADS ≥ 3 performance compared to fixed threshold adjustment, while the limit threshold-derived U-Net working point for PI-RADS ≥ 3 is nearly the same as for PI-RADS ≥ 3. See text for details. f3/d3/l3 = fixed/dynamic/limit threshold to match clinical performance at PI-RADS greater than or equal to 3; f4/d4/l4 = fixed/dynamic/limit threshold adjusted to match clinical performance at PI-RADS greater than or equal to 4. PI-RADS = Prostate Imaging Reporting and Data System

We denote U-Net performance according to fixed (f), dynamic (d), and limit (l) thresholds emulating PI-RADS ≥ 3 or ≥ 4 decisions in the form U-Net ≥ f3/d3/l3 and ≥ f4/d4/l4 respectively. The set of temporally distinct dynamic thresholds and the resulting performance metrics show small undulating fluctuations for d4 and a slow decrease for d3 over time as given in Fig. 2 and Table 2. A comparison of performance of PI-RADS and U-Net in new patients at different thresholds is given in Table 2. The distribution of biopsy results and referral indications in examinations influencing calculation of new dynamic thresholds d3 and d4 is given in Table 3, indicating no unexpected changes in referral indication or biopsy distribution. A direct comparison of stability and comparability of PI-RADS and dynamic threshold–adjusted U-Net performance in the look-back of 300 examinations is shown in Table 4. Using fixed thresholds, the patient-based working point U-Net ≥ f4 lies close to the PI-RADS ≥ 4 operating point (red diamond and triangle in Fig. 3a, respectively) with the corresponding fixed threshold (f4) of 0.31 being nearly equal to the limit threshold (l4) of 0.30 (Table 5), suggesting stability of the model. PI-RADS ≥ 3 and corresponding fixed threshold U-Net working point U-Net ≥ f3 are more distant from each other (green diamond and triangle in Fig. 3a, respectively) with the corresponding fixed threshold (f3) of 0.20 being different from the limit threshold (l3) of 0.14 showing that PI-RADS is better approximated using the dynamic threshold (d3) (green cross and triangle in Fig. 3a). A lack of deterioration in U-Net ROC discrimination in the new cohort is indicated by the blue ROC curve and the limit threshold-related working points (red (l4) and green (l3) circles) in Fig. 3a lying very close to the respective PI-RADS working points. In the sextant-based assessment, there is a strong improvement from U-Net ≥ f3 (green diamond in Fig. 3b) to U-Net ≥ d3 (green cross in Fig. 3b), while there is a small improvement from U-Net ≥ f4 (red diamond in Fig. 3b) to U-Net ≥ d4 (red cross in Fig. 3b). We thus utilize dynamic threshold adjustment for performance comparison to radiologists in the remaining model validation.

**Table 2:** Performance of PI-RADS and U-Net using different thresholds in simulated prospective assessment, reported per batch of 50 new examinations, with adjustment of dynamic thresholds (d3 and d4) occurring after each batch

Step	*n*	f3	d3	l3	PI-RADS ≥ 3 Sens | Spec	U-Net ≥ f3 Sens | Spec	U-Net ≥ d3 Sens | Spec	U-Net ≥ l3 Sens | Spec	f4	d4	l4	PI-RADS ≥ 4 Sens | Spec	U-Net ≥ f4 Sens | Spec	U-Net ≥ d4 Sens | Spec	U-Net ≥ l4 Sens | Spec
*0*	*50*	0.196	0.196	0.143	100% | 12%	94% | 39%	94% | 39%	100% | 15%	0.308	0.308	0.300	88% | 61%	76% | 70%	76% | 70%	76% | 70%
*1*	*50*	0.196	0.168	0.143	100% | 12%	100% | 40%	100% | 32%	100% | 24%	0.308	0.298	0.300	92% | 64%	80% | 68%	88% | 64%	88% | 64%
*2*	*50*	0.196	0.151	0.143	96% | 21%	96% | 29%	100% | 25%	100% | 21%	0.308	0.282	0.300	81% | 63%	81% | 58%	85% | 50%	85% | 58%
*3*	*50*	0.196	0.141	0.143	100% | 16%	100% | 45%	100% | 16%	100% | 16%	0.308	0.282	0.300	84% | 58%	95% | 61%	95% | 55%	95% | 61%
*4*	*59*	0.196	0.140	0.143	95% | 21%	86% | 24%	100% | 11%	100% | 11%	0.308	0.282	0.300	76% | 50%	62% | 42%	71% | 39%	71% | 42%

Abbreviations: *Step* threshold adjustment step index, *n* number of examinations for which metrics are calculated, *f3/d3/l3* fixed/dynamic/limit threshold to match clinical performance at PI-RADS greater than or equal to 3, *Sens* sensitivity, *Spec* specificity, *f4/d4/l4* fixed/dynamic/limit threshold adjusted to match clinical performance at PI-RADS greater than or equal to 4, *PI-RADS* Prostate Imaging Reporting and Data System

**Table 3:** Distribution of biopsy results and referral indication for look-back cohorts of 300 examinations utilized for the recalculating of dynamic thresholds at threshold readjustment steps occurring every 50 patients. For distribution of patients, see the 2nd and 3rd columns in Table 4

Threshold adjustment step	*n*	No PC (%)	ISUP grade group I (%)	ISUP grade group II (%)	ISUP grade group III (%)	ISUP grade group IV (%)	ISUP grade group V (%)	Number of patients with sPC (%)	Biopsy-naïve (%)	Active surveillance (%)	Previously biopsied (%)
0	300	120 (40)	65 (22)	76 (25)	16 (5)	11 (4)	12 (4)	115 (38)	140 (47)	79 (26)	81 (27)
1	300	118 (40)	69 (23)	76 (25)	15 (5)	9 (3)	13 (4)	113 (38)	146 (49)	78 (26)	76 (25)
2	300	121 (40)	66 (22)	73 (24)	14 (5)	12 (4)	14 (5)	113 (38)	155 (52)	76 (25)	69 (23)
3	300	118 (39)	57 (19)	77 (25)	17 (6)	14 (5)	17 (6)	125 (42)	156 (52)	73 (24)	71 (24)
4	300	121 (41)	57 (19)	73 (24)	18 (6)	13 (4)	18 (6)	122 (41)	157 (52)	74 (25)	69 (23)
5	300	118 (40)	58 (20)	76 (25)	19 (6)	10 (3)	19 (6)	124 (41)	160 (53)	72 (24)	68 (23)

Abbreviations: *PC* prostate cancer, *ISUP* International Society of Urological Pathology, *sPC* significant PC, *n* number of examinations

**Table 4:** Comparison of clinically achievable prediction agreement between dynamically adjusted U-Net and PI-RADS over time, reported on the sliding-window look-back of 300 examinations in 50 examination increments

Threshold adjustment step	*N*_p_ (prior cohort)	*N*_c_ (current cohort)	*n*	d3	PI-RADS ≥ 3 Sensitivity | specificity	U-Net ≥ d3 Sensitivity | specificity	d4	PI-RADS ≥ 4 Sensitivity | specificity	U-Net ≥ d4 Sensitivity | specificity
*0*	*300*	*0*	*300*	0.196	97% | 27%	97% | 26%	0.308	86% | 56%	84% | 56%
*1*	*250*	*50*	*300*	0.168	97% | 23%	97% | 23%	0.298	87% | 56%	84% | 56%
*2*	*200*	*100*	*300*	0.151	99% | 17%	98% | 17%	0.282	89% | 54%	88% | 53%
*3*	*150*	*150*	*300*	0.141	98% | 12%	99% | 12%	0.282	88% | 53%	86% | 53%
*4*	*100*	*200*	*300*	0.140	98% | 13%	99% | 13%	0.282	87% | 55%	85% | 54%
*5*	*41*	*259*	*300*	0.140	98% | 15%	99% | 15%	0.297	86% | 56%	83% | 56%

Abbreviations: *N*_*p*_ *(prior cohort)* number of most recent examinations from the original U-Net training cohort considered for each threshold adjustment step; *N*_*c*_ *(current cohort)* number of consecutive examinations from the current study cohort considered for each threshold adjustment step; at each step, *N*_p_ + *N*_c_ = 300 examinations were used to determine the new threshold; *n* number of men considered for sensitivity and specificity analysis; *d3* dynamic threshold adjusted to match clinical performance at PI-RADS greater than or equal to 3; *d4* dynamic threshold adjusted to match clinical performance at PI-RADS greater than or equal to 4; *PI-RADS* Prostate Imaging Reporting and Data System

**Table 5:** Diagnostic performance of clinical and U-Net assessment compared with combined targeted and systematic sextant biopsy histopathologic mapping on a per-patient and per-sextant basis, given for fixed, dynamic, and limit U-Net probability thresholds

*U-Net probability threshold*		Sensitivity	95% CI Sensitivity	Specificity	95% CI Specificity	PPV	NPV	*p* value Sens	*p* value Spec
Patient-based									
	PI-RADS ≥ 3	98% (106/108)	94.100	17% (25/151)	11.24	46% (106/232)	93% (25/27)	Ref	Ref
	PI-RADS ≥ 4	84% (91/108)	76.91	58% (88/151)	50.66	59% (91/154)	84% (88/105)	Ref	Ref
Fixed	UPT ≥ f3	95% (103/108)	90.99	35% (53/151)	28.43	51% (103/201)	91% (53/58)	–	–
	UPT ≥ f4	79% (85/108)	70.86	59% (89/151)	51.67	58% (85/147)	79% (89/112)	–	–
Dynamic	UPT ≥ d3	99% (107/108)	95.100	24% (36/151)	17.31	48% (107/222)	97% (36/37)	> 0.99	> 0.99
	UPT ≥ d4	83% (90/108)	75.90	55% (83/151)	47.63	57% (90/158)	82% (83/101)	> 0.99	> 0.99
Limit	UPT ≥ l3	100% (108/108)	97.100	17% (25/151)	11.24	46% (108/234)	100% (25/25)	–	–
	UPT ≥ l4	83% (90/108)	75.90	58% (88/151)	50.66	59% (90/153)	83% (88/106)	–	–
Sextant-based									
	PI-RADS ≥ 3	71% (177/251)	65.76	62% (814/1303)	60.65	27% (177/666)	92% (814/888)	Ref	Ref
	PI-RADS ≥ 4	63% (158/251)	57.69	80% (1045/1303)	78.82	38% (158/416)	92% (1045/1138)	Ref	Ref
Fixed	UPT ≥ f3	62% (155/251)	55.68	74% (967/1303)	72.77	32% (155/491)	91% (967/1063)	–	–
	UPT ≥ f4	47% (119/251)	41.54	85% (1113/1303)	83.87	39% (119/309)	89% (1113/1245)	–	–
Dynamic	UPT ≥ d3	70% (175/251)	64.75	66% (860/1303)	63.69	28% (175/618)	92% (860/936)	> 0.99	0.34
	UPT ≥ d4	51% (129/251)	45.58	84% (1096/1303)	82.86	38% (129/336)	90% (1096/1218)	0.01*	0.02*
Limit	UPT ≥ l3	73% (183/251)	67.78	62% (807/1303)	59.65	27% (183/679)	92% (807/875)	–	–
	UPT ≥ l4	49% (123/251)	43.55	85% (1105/1303)	83.87	38% (123/321)	90% (1105/1233)	–	–

Abbreviations: *UPT* U-Net probability thresholds; *f3/d3/l3* fixed/dynamic/limit threshold to match clinical performance at PI-RADS greater than or equal to 3; f3 = 0.20, d3 is dynamically adjusted (see text), l3 = 0.14; *f4/d4/l4* fixed/dynamic/limit threshold adjusted to match clinical performance at PI-RADS greater than or equal to 4; f4 = 0.31, d4 is dynamically adjusted (see text), l4 = 0.30; *PI-RADS* Prostate Imaging Reporting and Data System; *PPV* positive predictive value; *NPV* negative predictive value, *p* values (McNemar test) adjusted for multiple comparisons using Holm’s method

*Statistically significant

#### U-Net performance using dynamic thresholds

##### Patient-based performance

Clinical assessment had a sensitivity of 98% (106 of 108) and specificity of 17% (25 of 151) for PI-RADS category ≥ 3, and a sensitivity of 84% (91 of 108) and specificity of 58% (88 of 151) for PI-RADS category ≥ 4. U-Net had a sensitivity of 99% (107 of 108) and specificity of 24% (36 of 151, *p* > 0.99) for U-Net ≥ d3 and a sensitivity of 83% (90 of 108) and specificity of 55% (83 of 151, *p* > 0.99) for U-Net ≥ d4 (Table 5 and Fig. 3a).

##### Sextant-based performance

Clinical assessment had a sensitivity of 71% (177 of 251) and specificity of 62% (814 of 1303) for the PI-RADS ≥ 3, and a sensitivity of 63% (158 of 251) and specificity of 80% (1045 of 1303) for PI-RADS ≥ 4. U-Net had a sensitivity of 70% (175 of 251; *p* > 0.99) and specificity of 66% (860 of 1303; *p* = 0.34) for U-Net ≥ d3 and a sensitivity of 51% (129 of 251; significantly lower than PI-RADS; *p* = 0.01) and specificity of 84% (1096 of 1303; significantly higher than PI-RADS; *p* = 0.02) for U-Net ≥ d4 (Table 5 and Fig. 3b).

##### Co-occurrence of U-Net and PI-RADS assessment

Co-occurrent detection of men, sextants*,* and lesions by both U-Net and PI-RADS assessment at various thresholds is shown in Table 6. In individual men, with co-occurrent detection of PI-RADS ≥ 4 and *U-Net ≥ d3,* the positive predictive value (PPV) increased from 59% (91 of 154) to 63% (91 of 145; *p* = 0.03) with an insignificant increase of negative predictive value (NPV) from 84% (88 of 105) to 85% (97 of 114, *p* = 0.15). With co-occurrent detection of PI-RADS ≥ 4 and *U-Net ≥ d3* sextants*,* the PPV increased from 38% (158 of 416) to 47% (135 of 287; *p* = < 0.001) with an insignificant decrease of NPV from 92% (1045 of 1138) to 91% (1151 of 1267*;* *p* = 0.07). Co-occurrent detection of PI-RADS ≥ 4 and *U-Net ≥ d3* lesions increased the PPV from 43% (96 of 223) to 60% (87 of 145; *p* = < 0.001) with an insignificant decrease of NPV from 92% (181 of 197) to 91% (250 of 275*;* *p* > 0.99).

**Table 6:** Simultaneous detection of sPC in men, sextants, and lesions by PI-RADS and U-Net at given thresholds

			PI-RADS ≥ 3		PI-RADS ≥ 4	
	PPV	NPV	PPV	NPV	PPV	NPV
Patient-based						
			46% (106/232)	93% (25/27)	59% (91/154)	84% (88/105)
UPT ≥ d3	48% (107/222)	97% (36/37)	53% (105/199) [< 0.001]	95% (57/60) [> 0.99]	63% (91/145) [0.03]	85% (97/114) [0.15]
UPT ≥ d4	57% (90/158)	82% (83/101)	60% (89/148)	83% (92/111)	69% (81/118)	81% (114/141)
Sextant-based						
			27% (177/666)	92% (814/888)	38% (158/416)	92% (1045/1138)
UPT ≥ d3	28% (175/618)	92% (860/936)	39% (147/379) [< 0.001]	91% (1071/1175) [> 0.99]	47% (135/287) [< 0.001]	91% (1151/1267) [0.07]
UPT ≥ d4	38% (129/336)	90% (1096/1218)	46% (116/251)	90% (1168/1303)	51% (109/214)	89% (1198/1340)
Lesion-based						
			28% (112/403)	100% (17/17)	43% (96/223)	92% (181/197)
UPT ≥ d3	–	–	49% (92/188) [< 0.001]	91% (212/232) [< 0.001]	60% (87/145) [< 0.001]	91% (250/275) [> 0.99]
UPT ≥ d4	–	–	57% (73/128)	87% (253/292)	64% (69/107)	86% (270/313)

Abbreviations: *UPT* U-Net probability thresholds; *PI-RADS* Prostate Imaging Reporting and Data System; *d3* dynamic threshold adjusted to match clinical performance at PI-RADS greater than or equal to 3; *d4* dynamic threshold adjusted to match clinical performance at PI-RADS greater than or equal to 4; *PPV* positive predictive value; *NPV* negative predictive value, *p* values in brackets (DTComPair R package) adjusted for multiple comparisons using Holm’s method

#### Spatial congruence of segmentations

**Fig. 4:**
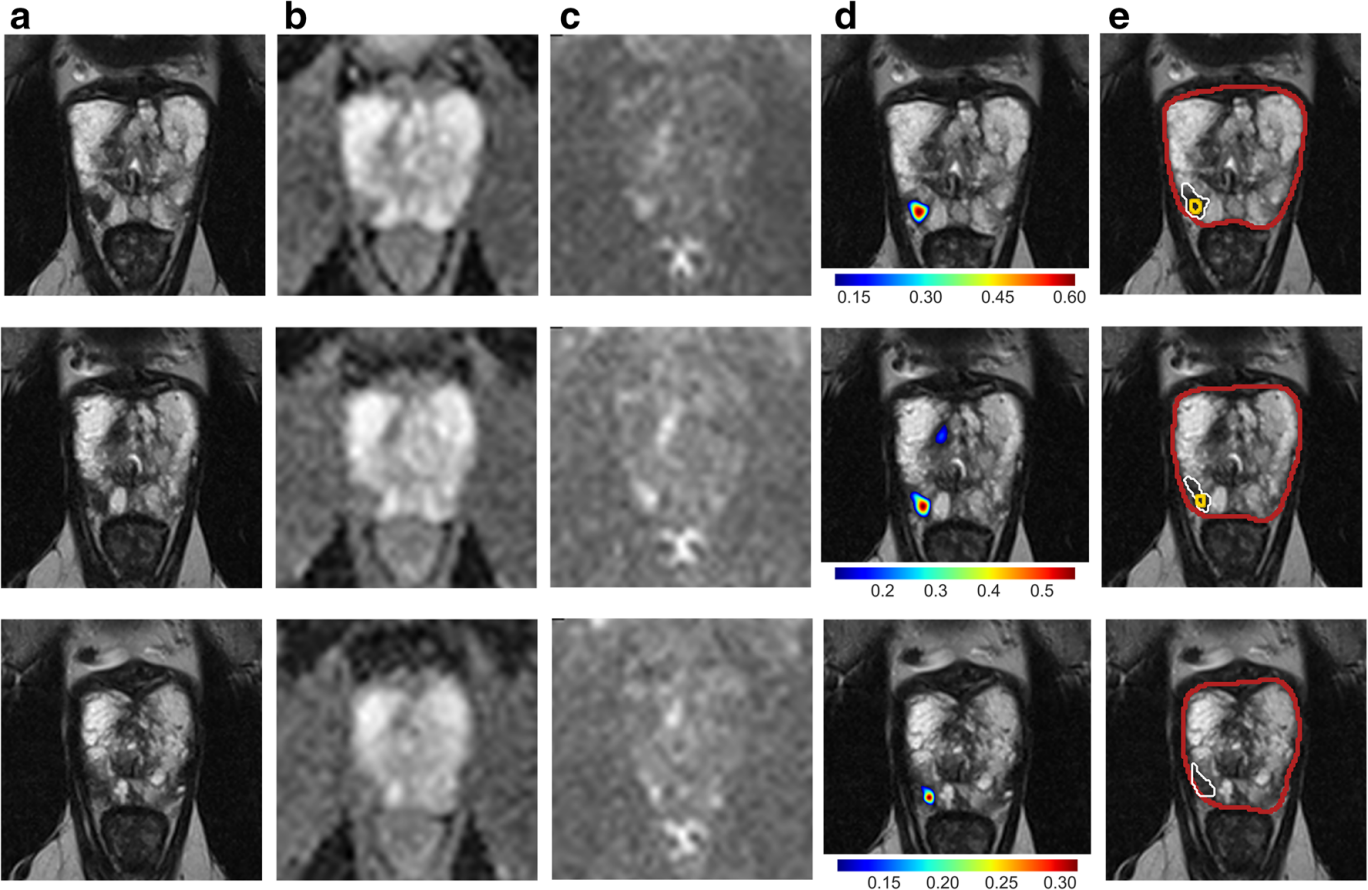
Images show examples of U-Net ensemble segmentation in a 59-year-old man with PSA of 4.4 ng/ml and Prostate Imaging Reporting and Data System category 4 lesion (Gleason grade group 2) in right posterior peripheral zone on three consecutive MRI slices (rows). **a** T2w, (**b**) apparent diffusion coefficient, and (**c**) *b*-value 1500 s/mm^2^ images (apparent diffusion coefficient and *b*-value 1500 s/mm^2^ images registered using rigid followed by b-spline registration). **d** T2w image (grayscale) overlaid with U-Net ensemble output probability map for the tumor class (color scale). **e** T2w image with overlaid segmentations: U-Net-derived prostate segmentation in red, hand-drawn clinical T2w lesion segmentation in white, and U-Net ensemble-derived tumor lesion segmentation in yellow. The tumor dice score was 0.12, 0.12, and 0.08 for DWI, T2w, and combined, respectively. The maximum tumor probability predicted by the U-Net ensemble was 0.61. PI-RADS = Prostate Imaging Reporting and Data System

**Fig. 5:**
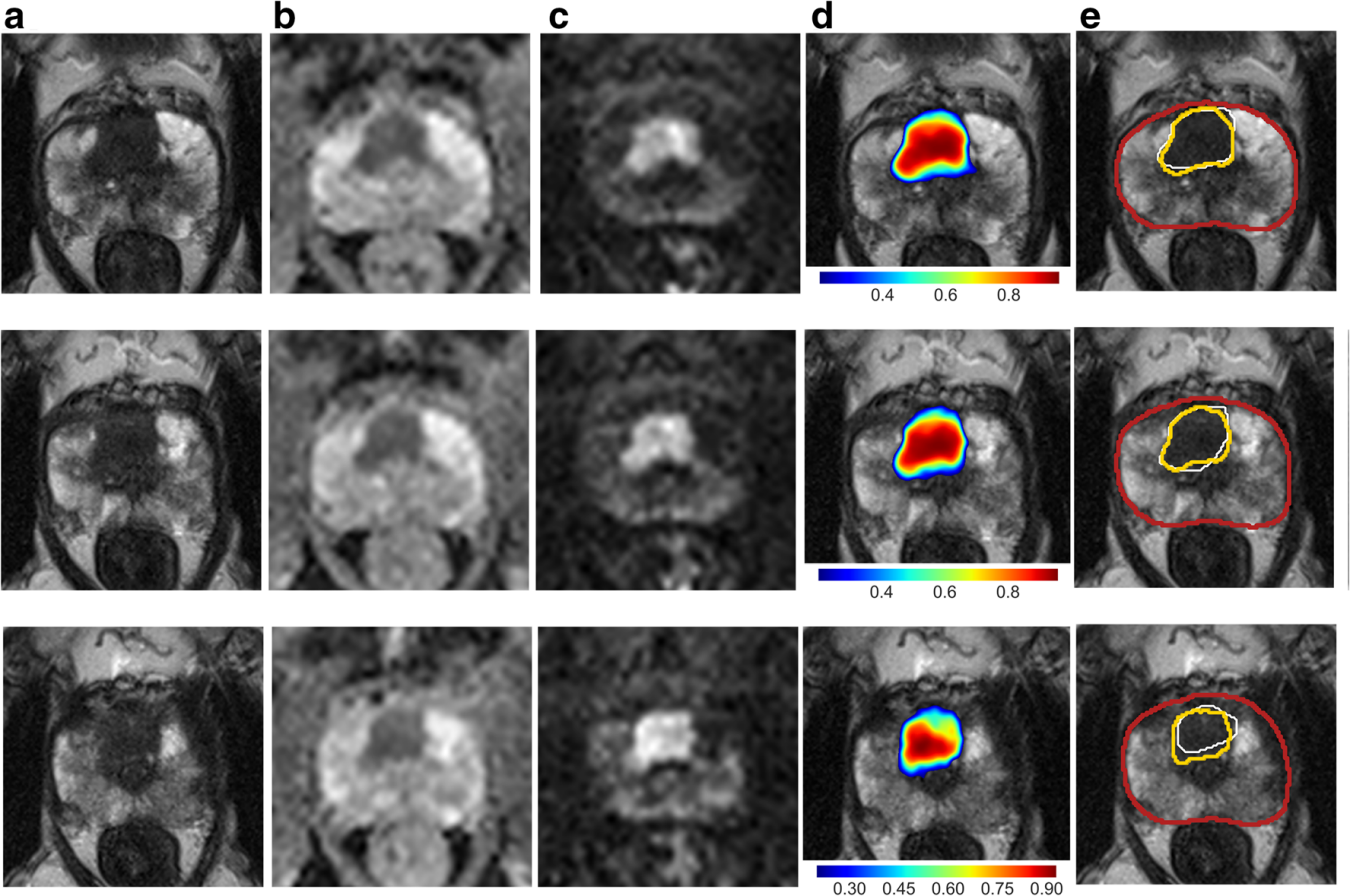
Images show example of U-Net ensemble segmentation in a 71-year-old man with PSA of 9.4 ng/ml and Prostate Imaging Reporting and Data System category 5 lesion (Gleason grade group 2) extensively in the anterior transition zone bilaterally on three consecutive MRI slices (rows). **a** T2w, (**b**) apparent diffusion coefficient, and (**c**) *b*-value 1500 s/mm^2^ images (apparent diffusion coefficient and *b*-value 1500 s/mm^2^ images registered using rigid followed by b-spline registration). **d** T2w image overlaid with U-Net ensemble output probability map for the tumor class. **e** T2w image with overlaid segmentations: U-Net-derived prostate segmentation in red, hand-drawn clinical T2w lesion segmentation in white, U-Net ensemble-derived tumor lesion segmentation in yellow. The tumor dice score was 0.72, 0.55, and 0.58 for DWI, T2w, and combined, respectively. The maximum tumor probability predicted by the U-Net ensemble was 0.96. PI-RADS = Prostate Imaging Reporting and Data System

Dice coefficient of targeted sPC-positive PI-RADS lesion segmentations and U-Net*-*derived lesion segmentations was 0.34, 0.34*,* and 0.29 for DWI, T2w*,* and the combination, respectively. Dice coefficient distributions are shown in Supplementary Fig. 1 for overlapping lesion segmentations. Figures 4 and 5 demonstrate representative examples of U-Net output compared with manual segmentations.

## Discussion

Prostate MRI is increasingly incorporated into the standard diagnostic pathway. Deep learning carries potential to disseminate high-quality prostate MRI assessment and support image interpretation while demand for prostate MRI increases. This study represents the first simulation of clinical deployment of a validated deep learning system for fully automatic prostate MRI assessment within the clinical environment for which it was optimized. Clinical deployment is simulated as it is still too early to actually deploy this system into prospective clinical practice affecting clinical decisions, while the simulated analysis provides important information on what performance can be expected. Comparable performance to clinical MRI assessment was confirmed, i.e., sensitivity 84% [91/108] vs. 83% [90/108], *p* > 0.99; specificity 58% [88/151] vs. 55% [83/151], *p* > 0.99, respectively, for PI-RADS ≥ 4 vs. dynamic U-Net threshold. By simulating continued clinical application of deep learning in consecutive patients, the stability over defined periods of operation and the effect of readjustment of the system with respect to PI-RADS could be closely evaluated. The achieved degree of assessment of model fitness for clinical application thus is much advanced in comparison to explorative studies of deep learning performance [23–25]. Using a quality assurance cycle of 50 patients or approximately two months, we find that fluctuations between PI-RADS and U-Net performance can be reduced by a recalibration scheme which, when used prospectively, assures similar performance of both assessment methods. These fluctuations were minor for PI-RADS ≥ 4 decisions and the diagnostic performance stable over the 300 examination look-back period. However, a slow decrease of d3 and the specificity of PI-RADS *≥* 3 decisions in the look-back period with otherwise congruence of the U-Net ROC curve and the PI-RADS operating points in the new cohort suggests that the difference is neither caused by a deterioration of the system (as the U-Net ROC curve is very close to the PI-RADS working points) nor a drift in the composition of tumors in the cohort (cf. Table 3) or the image quality (scanner and image protocol remained the same), but rather related to a shift in PI-RADS interpretation. While the composition of the team of radiologists changed slightly since the previous cohort, the isolated change at PI-RADS *≥* 3 suggests that this is of minor importance, such that this finding may be explained by the PI-RADS 3 category being the least clearly defined (the “indeterminate”) category of the system. It is subject to ongoing re-definition and by nature includes subtle and nonspecific lesions which may be evaluated differently by a team of radiologists over time. In a sense, U-Net at fixed thresholds can be compared to an isolated radiologist or team of radiologists performing assessments without being integrated into any ongoing case reviews and communication with the team of radiologists that contributed to its initial training. It may be the case that radiologists make joint decisions resulting from clinical feedback and case conferences that adjust PI-RADS 3 reading patterns slightly toward more specific or sensitive reporting style, depending on the agreed-upon direction of continued quality improvement. The same may be observed for a team of radiologists that splits in two and ceases communication. To decide which of either a) the rigid performance of fixed U-Net thresholds (which still provide clinically reasonable working points and may represent the advantage of artificial intelligence to reduce inter-rater variability) or b) the dynamic response of the radiologists (which represents continuous situation-aware learning) is better requires more investigation in the future. At the moment, we observe one system (U-Net) which has ceased learning (fixed thresholds) compared to one that continues to learn from clinical practice (radiologists). Still, with radiologists being certified for clinical practice while U-Net is not, PI-RADS lends itself to be used as standard, with dynamic threshold adjustment being identified as the method to effectively impose the same adjustments onto U-Net that the radiologists are making. The proposed threshold adjustment scheme gives flexibility for comparison and clinical implementation. When PI-RADS is used as “manual” input for calibration, the result is a semi-automatic calibration. One could, however, also use acceptable sensitivity ranges for calibration which would lead to an entirely data-driven, fully self-calibrating system.

A specific advantage of the cohort in our study is the analysis of consecutive at-risk patients, allowing a direct and clinically meaningful comparison of performance. In addition, the used extended systematic and targeted biopsies provide a much better assessment than standard sampling schemes having a sensitivity of up to 97% for sPC compared with radical prostatectomy (RP) [15]. In comparison, pure RP cohorts would introduce bias excluding many men that received MRI-guided biopsies but did not undergo RP; thus, the selected reference standard of extended systematic and targeted biopsies is of high quality for complete assessment of the population.

There are limitations to our study. The developed U-Net in its current form is applicable only to data from our main institutional MRI system. While it is desirable to develop more general AI systems in the future, the current system is expected to maximize the utility of deep learning at current still limited cohort sizes by avoiding added heterogeneity of multi-scanner cohorts which would require more data for equally successful machine learning. In the future, we plan to apply the developed U-Net in a prospective setting at our institution and to perform transfer learning on multi-centric data to expand its domain.

In conclusion, this study provides the first simulated clinical deployment of a previously validated AI system for fully automatic prostate MRI assessment. By simulating regular quality assurance cycles, we find that the system performance is stable for PI-RADS ≥ 4 decisions, while slowly changing clinical PI-RADS ≥ 3 assessment can be addressed by a newly proposed threshold adjustment scheme. Observed fluctuations may be an indication that deep learning can address inter-observer variability of PI-RADS or indicate the detachment of U-Net from the ongoing clinical quality assurance cycle with U-Net being re-attached by the proposed dynamic adjustment scheme. Co-occurrent detection by U-Net and radiologists increased the probability of finding sPC. U-Net confirms itself as a powerful tool to extract a diagnostic assessment from prostate MRI and its performance motivates evaluation in a prospective setting.

## Electronic supplementary material

ESM 1 (DOCX 324 kb)

## Abbreviations

ADC: Apparent diffusion coefficient
DCE-MRI: Dynamic contrast-enhanced (DCE) MRI
ISUP: International Society of Urological Pathology
nPT: Normal-appearing prostate tissue
NPV: Negative predictive value
PI-RADS: Prostate Imaging Reporting and Data System
PPV: Positive predictive value
PSA: Prostate-specific antigen
ROC: Receiver operating characteristics
RP: Radical prostatectomy
sPC: Clinically significant prostate cancer
T2w: T2-weighted
UPT: U-Net probability thresholds
VOI: Volume of interest
