# Supplementary material for: Simulated clinical deployment of fully automatic deep learning for clinical prostate MRI assessment
Source: Eur Radiol. 2020 Aug 7;31(1):302–13. doi: 10.1007/s00330-020-07086-z (PMC7755653; doi:10.1007/s00330-020-07086-z)
Supplement: Supplementary file 1 — (DOCX 324 kb) [file 330_2020_7086_MOESM1_ESM.docx]

Supplementary Material

Simulated Clinical Deployment of Fully-Automatic Deep Learning for Clinical Prostate MRI Assessment

1. *Image Pre-processing and Image Registration*

Images were preprocessed and registered with the SimpleITK toolbox [27; 28] and its extension SimpleElastix (https://simpleelastix.github.io) [29]. Co-registration employed the Mattes Mutual Information criterion [30] and utilized three different approaches: (a) rigid registration, (b) rigid followed by affine registration and (c) rigid followed by b-spline registration, to enhance the detection of small lesions, hypothesizing that one of these approaches would best superimpose small lesions on both sequences. The DICOM images for the T2-weighted, and diffusion-weighted images were converted to the nrrd format. The resolution of the diffusion weighted images was up-sampled to the T2-weighted image resolution 0.3125x0.3125x3 mm and zero-padded when the up-sampled diffusion-weighted image had a smaller size than the T2-weighted image or cropped if the respective image was of greater size. The T2-weighted image was used as reference while the apparent diffusion coefficient image was used as the moving sequence. Images were registered first using EulerTransform with the initial settings from the default parameter map “rigid” from the SimpleElastix toolbox. For the registration process we increased the “NumberOfSpatialSamples” and the “NumberOfSpatialSamplesForExactGradient” to 256000. We used 150 “NumberOfHistogramBins” and 512 “MaximumNumberOfIterations” for all registration schemes. The final rigid registration transform was applied to the b-value 1500 sec/mm2 image. After initial registration all images were cropped to a matrix size of 320x320 and registered using the “affine” and the “bspline” parameter map from the SimpleElastix toolbox. For the affine registration we used 256000 “NumberOfSpatialSamples” and 256000 “NumberOfSpatialSamplesForExactGradient”. For the bspline registration we used 128000 “NumberOfSpatialSamples” and 128000 “NumberOfSpatialSamplesForExactGradient”; a “FinalGridSpacingInPhysicalUnits” of 4 and a “Metric1Weight” of 250. Rigid registered images were cropped to a matrix size 320x320 and all images, including the affine and b-spline registered images down sampled to a resolution of 0.625x0.625x3mm before prediction by the U-Net ensemble. In our previous approach registration depended on manual prostate segmentation for sampling of reference points during affine registration. The current approach samples reference points from the whole image data, now rendering the registration fully automatic.

1. *Combined Ground Truth Map and Lesion Evaluation*

The combination of sextant-specific biopsy information and targeted lesion histopathology have previously been shown to have high agreement with radical prostatectomy (RPE) specimen [26]. Both were fused into a combined ground truth map by additionally considering all sextants as positive whose automatically generated segmentations from U-Net prostate segmentations and the geometric definition of the Ginsburg biopsy scheme [11; 31; 32] overlapped at least one voxel with sPC-positive segmentations of biopsy targets. U-Net derived lesions were defined as those voxels where the probability for the tumor class was higher than the sum of the normal appearing prostate class probability and the background class probability. When evaluating manual and U-Net-derived segmentations against the fused ground truth, to limit the influence of lesions only partially overlapping with sextant boundaries only these sextants were considered to contain a lesion when at least 50% of the lesion was occupied by the respective sextant or at least half of the sextant was occupied by the lesion.

1. *Selection of U-Net probability cut-off values*

U-Net tumor probability thresholds were chosen from the patient-based ROC curve such that they most closely matched performance of the clinical radiologist at PI-RADS cut-offs ≥3 and ≥4. Thresholds were selected by calculating the absolute difference in the True Positive Rate (TPR) and the False Positive Rate (FPR) between the ROC probability cut-offs and the evaluated PI-RADS working points. Those tumor probability thresholds were chosen where the sum of the calculated TPR and FPR difference compared to the PI-RADS working points was smallest.

1. *Python Packages*

We used Python 3.7.3 as programming language for analysis, data preparation and visualization. Packages used in the SciPy [33] ecosystem included: Pandas 0.24.2 [34], Numpy 1.16.4 [35], scikit-learn 0.21.2 [36], Matplotlib 3.1.0 [37], Seaborn 0.9.0. [38], Statsmodel 0.10.0 [39] was used for McNemar test calculation. Statistical calculation was partly done in R version 3.6.0. For confidence interval calculation the R package ModelGood 1.0.9 was used. Correction for multiple comparison was calculated with the R package stats 3.6.0 using Holm´s method.

1. *Full cohort dice coefficient calculation*

All voxels from biopsy sPC positive clinical lesion segmentations and U-Net derived voxels with the tumor class probability being greater than the sum of the normal appearing prostate class probability and the background class probability were pooled and considered true positive (TP), if voxels from both masks where congruent, false positive (FP), if U-Net mask voxels where outside and false negative (FN), if negative inside biopsy positive clinical lesions. Dice coefficient was calculated for all pooled voxel data according to the formula: DSC = 2TP / (2TP + FP + FN).

**Supplementary Table 1:**

| **Prostate MRI protocol** | | | | | | | | | | |
| --- | --- | --- | --- | --- | --- | --- | --- | --- | --- | --- |
| Sequence | Orientation | TE (ms) | TR (ms) | Slice thickness (mm) | FOV read (mm) / FoV phase % | Base /phase %/ (slice %) Resolution | Phase encoding direction | Averages | Additional features | Additional features |
| T2 blade | sag | 105 | 5000 | 3 | 200 / 100% | 320 / 100% | F-H | 1 | Blade coverage 81% |  |
| T1 tse dixon | cor | 13 | 773 | 4 | 360 / 90% | 320 / 100% | F-H | 1 | TF 5 |  |
| T2 tse | tra | 145 | 8080 | 3 | 200 / 100% | 320 / 100% | R-L | 3 | TF 27 |  |
| T2 tse | cor | 145 | 8000 | 3 | 200 / 100% | 320 / 80% | F-H | 3 | TF 19 |  |
| epi_diff SPAIR | tra | 48 | 3300 | 3 | 280 / 74 % | 140 / 100% | A-P | 2,2,4,6 | phase partial Fourier 7/8 | b=50/500/ 1000/1500 |
|  |  |  |  |  |  |  |  |  |  |  |
| dyn DCE |  |  |  |  |  |  |  |  |  |  |
| T1_twist_DCE_dyn | tra | 2,1 | 4,45 | 3 | 300 / 87 % | 256 /85% / 65% | R-L | 1 | 35 meas. / Temporal Resolution 5 sec | 30 slices p.slab |
|  |  |  |  |  |  |  |  |  |  |  |
| T1_vibe_dixon | tra | 2,4 / 3,69 | 5,4 | 1.2 | 380 / 68 % | 384 / 80% / 50% | A-P | 1 | W-images / F-images | 224 slices p. slab |
| T1_tse_dixon | cor | 13 | 773 | 4 | 360 / 90% | 320 / 100% | F-H | 1 | TF 5 |  |
| General preparations: MRI-coil: body array coil; Patients placed supine; | | | | | | | | | | |
| *TE= echo time, TR = repetition time, FOV = Field of view; TSE = turbo-spin-echo; DCE = dynamic contrast enhanced; SPAIR= spectral adiabatic inversion recovery; FOV = field of view, TF = turbo factor, tra/sag/cor = transverse/sagittal/coronal* | | | | | | | | | | |


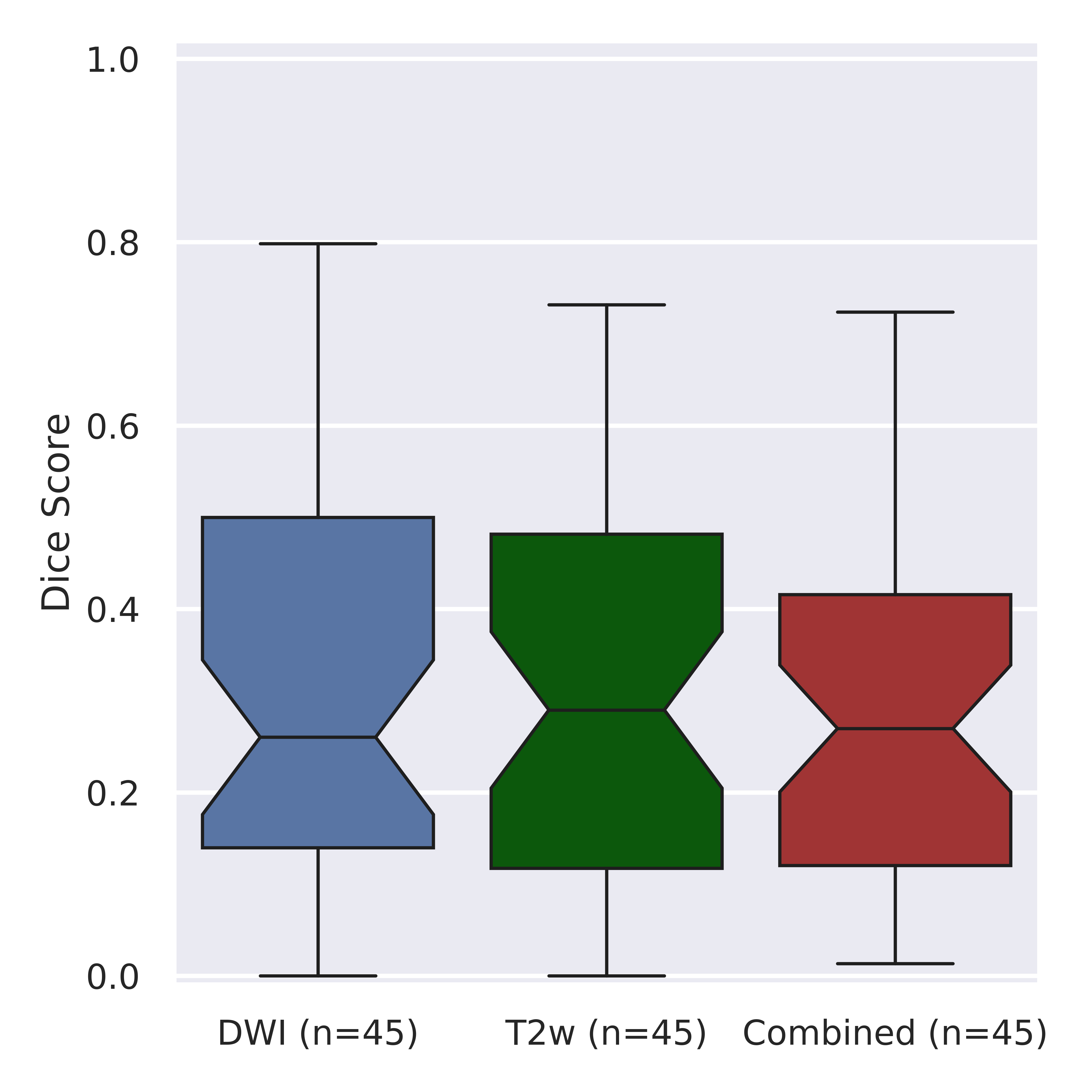
**Supplementary Figure 1** Notched box plots show Dice coefficient between targeted clinically significant prostate cancer (sPC) positive Prostate Imaging Reporting and Data System (PI-RADS) lesion segmentations and U-Net derived lesion segmentations on a per patient basis. Dice coefficient ranges between 0 and 1, with 0 meaning no overlap at all and 1 meaning identical lesions. U-Net generated segmentations were compared against hand-drawn DWI lesion segmentations (left, blue), T2w lesion segmentations (middle, green) and DWI with T2w combined lesion segmentations (AND operator) (right, red). Only Dice coefficients of patients with overlapping lesion segmentations in the combined setting are shown. The whiskers length is limited to a maximum of 1.5 times the interquartile rang. Notches indicate the 95% confidence interval around the median.
